# Immunobiological effects of tocilizumab across respiratory subphenotypes in COVID-19 ARDS

**DOI:** 10.1186/s40635-025-00779-z

**Published:** 2025-07-09

**Authors:** Emma Rademaker, Daan F. L. Filippini, Jelle L. G. Haitsma Mulier, Marleen A. Slim, Rombout B. E. van Amstel, Sivasubramanium V. Bhavani, Nicole P. Juffermans, Harm-Jan S. de Grooth, Lennie P. G. Derde, Olaf L. Cremer, Lieuwe D. J. Bos, Amsterdam U. M. C. COVID-19 Biobank Study Group

**Affiliations:** 1 Department of Intensive Care Medicine, UMC Utrecht, Utrecht University, Heidelberglaan 100, 3584 CX Utrecht, The Netherlands; 2 Julius Center for Health Sciences and Primary Care, Utrecht University, Utrecht, The Netherlands; 3 Department of Intensive Care Medicine, Amsterdam UMC, University of Amsterdam, Amsterdam, The Netherlands; 4 Department of Medicine, Emory University, Atlanta, Georgia; 5 Laboratory of Translational Intensive Care, Erasmus MC, University Medical Center, Rotterdam, The Netherlands; 6 Department of Intensive Care, Erasmus Medical Center, Rotterdam, The Netherlands; 7 Department of Pulmonology, Amsterdam UMC, University of Amsterdam, Amsterdam, The Netherlands; 8 Laboratory of Experimental Intensive Care and Anaesthesiology (L.E.I.C.A.), University of Amsterdam, Amsterdam, The Netherlands

## Abstract

**Background:**

Two distinct longitudinal respiratory subphenotypes have recently been described in COVID-19-related acute respiratory distress syndrome (ARDS). These subphenotypes exhibit dynamic immunobiological changes that may help guide immunomodulatory interventions. However, the extent to which the immune response is determined by respiratory subphenotype in the presence of concurrent immunomodulatory treatment remains unclear. We investigated the independent and combined effects of respiratory subphenotype and tocilizumab on inflammatory response and clinical outcomes.

**Methods:**

We analyzed patients from existing COVID-19 biobanks who were consecutively admitted to the ICU and received more than 4 days of invasive mechanical ventilation between March 2020 and May 2022. Patients were classified into two previously described longitudinal respiratory subphenotypes—characterized by mechanical power, minute volume and ventilatory ratio—referred to as ‘*low-power*’ and ‘*high-power*’ subphenotypes. We analyzed how tocilizumab treatment and respiratory subphenotype were associated with endothelial and inflammatory plasma biomarkers on days 0, 4 and 7, as well as with mortality.

**Results:**

720 patients were included, of whom 464 (64%) and 256 (36%) were assigned to the low- and high-power subphenotypes, respectively. 108 (23%) of the low-power subphenotype patients received tocilizumab, and 43 (17%) of the high-power subphenotype. 427 patients had plasma samples available. The high-power subphenotype was associated with slightly higher SP-D, thrombomodulin and TNF-RI plasma concentrations on the day of intubation compared to the low-power subphenotype, along with a more rapid increase in IL-6 and TNF-RI levels in subjects who had received tocilizumab treatment (*β* = 0.14 log ng/ml, *p* = 0.022, and *β* = 0.06 log ng/ml, *p* = 0.014, respectively). Tocilizumab treatment accounted for four times more variance in IL-6 and angiopoietin-2 levels than subphenotype, while subphenotype explained only a small proportion of the variance and slightly more than tocilizumab for TNF-RI and thrombomodulin. Subphenotype did not modify the association between tocilizumab and mortality (IPTW adjusted hazard ratio 1.18; 95%CI 0.60–2.33).

**Conclusion:**

Respiratory subphenotypes showed varying TNF-RI and IL-6 responses to tocilizumab, but these differences were only minor compared to the drug’s overall immunobiological effect. This suggests that respiratory subphenotype should not determine tocilizumab treatment decisions.

**Supplementary Information:**

The online version contains supplementary material available at 10.1186/s40635-025-00779-z.

## Introduction

Acute respiratory distress syndrome (ARDS) is a critical complication frequently seen in the intensive care unit (ICU), resulting from either direct lung injury or systemic inflammation. It is characterized by endothelial and epithelial damage, excessive inflammation and coagulation disturbances [1, 2]. Immunomodulation has been explored as a promising treatment strategy, but the biological heterogeneity of ARDS has made it challenging to identify consistent treatment effects [3]. This has led to increasing interest in a (sub)phenotype-targeted approach that might tailor therapies to specific patient subgroups [4].

In COVID-19-related ARDS, two respiratory subphenotypes have been identified, each following distinct trajectories of mechanical power and ventilatory ratio over the first few days of mechanical ventilation, which were prognostic for the duration of ventilation and mortality, and show a differential response to PEEP/FiO2 strategy [5]. Pulmonary immunothrombosis is an important contributor to ineffective ventilation [6], and may be a driving force in the progressive development of the high mechanical power and ventilatory ratio subphenotype. Dysregulation of the immune response with endothelial dysfunction is not stable over time and varies significantly between patients [7]. Immunomodulatory treatment may drive some of these changes, possibly creating an interaction between respiratory subphenotype, immune response dynamics, and immunomodulation which is not yet studied.

To address this, we measured immunobiological patterns in critically ill patients with COVID-19-related ARDS. We hypothesized that the respiratory subphenotype progressing to high mechanical power and ventilatory ratio is associated with greater endothelial damage and inflammation and that these differences persist despite tocilizumab (IL-6 receptor blockade) treatment. Lastly, we expected a greater immunobiological response to tocilizumab in this subphenotype.

## Methods

### Study design and population

In this retrospective cohort study, we used Electronic Health Record (EHR) data and plasma samples that were sourced from two academic hospitals in The Netherlands: Amsterdam University Medical Center (AUMC) and University Medical Center Utrecht (UMCU). The requirement for informed consent for the use of clinical data was waived (Institutional Review Board number (IRB) UMCU 22-591, applicable to both centers). Plasma samples were sourced from two biorepositories: the AUMC COVID-19 Biobank study (IRB 2020_065), and the Molecular Diagnosis and Risk Stratification of Sepsis (MARS) biorepository (TCBio 23-296).

Patients consecutively admitted to the ICU were eligible for inclusion if they met the following criteria: (1) COVID-19 as the primary admission diagnosis, confirmed by a positive polymerase chain reaction test from a nasopharyngeal swab or tracheal aspirate, and (2) receiving invasive mechanical ventilation for at least 4 days. Patients were excluded if they underwent extracorporeal membrane oxygenation during this timeframe, as this interfered with the reliable measurement of respiratory parameters.

### Data collection and outcomes

Highly granular data were directly exported from the EHR to capture 8-hourly respiratory parameters, from intubation up to 96 h (see Supplementary methods, Figures S1–2), along with baseline clinical characteristics, details of immunomodulatory treatment and outcomes.

Treatment status was categorized in two groups: tocilizumab administration and no tocilizumab administration. Tocilizumab (IL-6 receptor blockade) was given as a single 8 mg/kg dose (max 800 mg). Corticosteroid regimens were not considered when creating these treatment groups.

The primary outcome of our study were longitudinally measured biomarker concentrations. To assess whether the effect of tocilizumab differed across subphenotypes, we evaluated 90-day mortality.

### Sample preparation and protein assays

Leftover daily ethylenediaminetetraacetic acid (EDTA)- and lithium-heparin plasma was stored within 6-h of blood draw at − 80 °C. Plasma samples were retrieved from the following three timepoints relative to the initiation of invasive mechanical ventilation: within 24 h (T0), day 3–5 (T4) and day 6–8 (T7). Biomarkers were selected based on their established roles as markers of endothelial damage, epithelial damage and inflammation. Angiopoietin-2, interleukin (IL)-6, surfactant protein-D (SP-D), tumor necrosis factor receptor-1 (TNF-RI), thrombomodulin and vascular cell adhesion molecule-1 (VCAM-1) were measured using a Luminex 6-plex assay (R&D Systems Inc., Minneapolis, United States; Service provider: Arcadia, UMCU, the Netherlands). Samples were randomized across plates and intra- and inter-assay coefficients of variance were obtained to assess the quality of measurements (Table S1.3.3). Values outside the detection limit were extrapolated using the standard curve or, if not possible, replaced by 0.5 times the lower limit of detection or the maximum of extrapolated values (Table S1.3.4).

### Subphenotype classification

Patients were classified into two previously identified respiratory trajectory subphenotypes—characterized by increasing trajectories of mechanical power, minute volume ventilation and ventilatory ratio—referred to as ‘*low-power*’ and ‘*high-power*’ subphenotypes [5]. To classify patients, we applied the original latent class model parameters, which incorporated 8-hourly respiratory data collected up to day four of invasive mechanical ventilation [5]. Probabilities of class membership were calculated, and patients were assigned to a subphenotype based on a probability cut-off of 0.5.

### Statistical analysis

Trajectories of the six biomarkers measured at T0, T4, and T7, and their interaction with subphenotype and tocilizumab use were evaluated using mixed-effects models. Biomarker trajectories were modeled with and without a quadratic term for time and with and without random effects for the slopes and intercepts to determine the best-performing model. Model performance was compared using the likelihood ratio test and Akaike’s Information Criterion (AIC). Restricted Maximum Likelihood (REML) was used to obtain unbiased effect estimates for the final models. Nakagawa’s marginal R^2^ was used to quantify the proportion of variance in biomarker trajectories explained by subphenotype and tocilizumab.

To explore the complex relationship between subphenotypes, tocilizumab and immunobiology, we applied multivariate Mixed Graphical Modelling separately at T0, T4 and T7. These models capture conditional dependencies in the data, making them well-suited for determining whether biomarker patterns are attributable to subphenotypes, tocilizumab or both. We fit the models using tenfold cross-validation for lambda selection for penalization and included only two-way interactions.

To assess whether the effect of tocilizumab on outcomes differed across subphenotypes, we used Cox proportional-hazards regression with both exposure adjustment and outcome adjustment for possible confounders between those who received tocilizumab and those who did not [8]. Exposure adjustment was done with inverse probability of treatment weighting (IPTW) based on a propensity score model that included demographic factors, clinical information, laboratory measurements, admission site, and ICU admission calendar date. Outcome adjustment was done by adding the variables with remaining baseline imbalance as covariates in the weighted outcome model. An interaction term between tocilizumab and subphenotype was added to evaluate effect modification.

Continuous data were reported as mean and standard deviation or median and interquartile range according to the distribution. Categorical data were presented as numbers with percentages. Between-group differences were assessed using a two-sample t-test, Wilcoxon signed-rank test or Chi-squared test as appropriate. A two-sided *p*-value of 0.05 was considered statistically significant. All data analyses were performed using R version 4.4.0. through the R-studio interface.

## Results

### Study population

720 patients were included in this study (Figure S3). The median age was 63 years (IQR: 56–71), and 490 (68%) were male (Table 1). 464 (64%) patients were classified to the ‘low-power’ subphenotype, and 256 (36%) to the ‘high-power’ subphenotype. The high-power subphenotype patients were characterized by increased trajectories of mechanical power, minute volume and ventilatory ratio (Figure S4). Tocilizumab had been administered more frequently to patients later classified as low-power rather than high-power subphenotype (23% vs. 17%; *p* = 0.06), with the difference approaching statistical significance.

**Table 1:** Patient characteristics

	Low-power subphenotype	High-power subphenotype	*P* value
*n*	464	256	
Demographics			
Age, mean (SD)	62 (11)	63 (10)	0.36
Male (%)	281 (61)	209 (82)	< 0.001
BMI, mean (SD)	30.3 (22.4)	28.6 (6.9)	0.35
Wave^a^			
1 (%)	102 (22)	72 (28)	< 0.01
2 (%)	125 (27)	81 (32)	
3 (%)	113 (24)	66 (26)	
4 (%)	124 (27)	37 (15)	
Comorbidities			
Cardiovascular insufficiency (%)	5 (1)	3 (1)	1.000
Chronic respiratory insufficiency (%)	7 (2)	9 (4)	0.14
COPD (%)	37 (8)	24 (9)	0.61
Diabetes (%)	121 (26)	59 (23)	0.42
Immuno-insufficiency (%)	61 (13)	29 (11)	0.56
Malignancy (%)	24 (5)	23 (9)	0.07
Subphenotypes^b^			
Hypoinflammatory (%)	213 (95) (*n* = 225)	128 (96) (*n* = 134)	0.91
Hyperinflammatory (%)	12 (5) (*n* = 225)	6 (4) (*n* = 134)	
Immunomodulation			0.02
None (%)	86 (19)	66 (26)	
Corticosteroids alone (%)	269 (58)	144 (56)	
Tocilizumab plus corticosteroids (%)	108 (23)	43 (17)	
Tocilizumab administration^c^			0.25
Prior to mechanical ventilation (%)	42 (81)	19 (79)	
1–2 days post-intubation (%)	4 (9)	5 (21)	
Disease severity at intubation^d^			
Vasopressin (%)	258 (64)	137 (62)	0.71
SOFA score	7 [5, 8]	7 [5, 8]	0.68
C-reactive protein (mg/L)	102 [44, 163]	140 [55, 246]	< 0.001
White blood cell count × 10⁹/L	10.0 [7.3, 13.3]	11.8 [8.6, 16.0]	< 0.01
Ventilation^e^			
Mechanical power (J/min)	21.9 [17.6, 26.5]	27.8 [22.7, 33.3]	< 0.001
Minute ventilation (L/min)	9 [7.8, 10.3]	11.1 [9.6, 12.4]	< 0.001
Ventilatory ratio	1.65 [1.40, 1.97]	2.16 [1.85, 2.56]	< 0.001
Driving pressure (cmH_2_O)	13 [11, 16]	14 [12, 17]	< 0.01
CO_2_ difference (mmHg)	9 [5, 13]	14 [9, 20]	< 0.001
PaO_2_/FiO_2_	142 [111, 177]	140 [111, 174]	0.71
Compliance (mL/cmH_2_O)	30 [24, 40]	32 [25, 41]	0.24
pH	7.37 [7.30, 7.41]	7.33 [7.26, 7.39]	< 0.001

Data are presented as medians [IQR: interquartile range], unless otherwise specified by ‘mean, (SD)’ indicating mean and standard deviation or (%) indicating numbers and percentages. (a) Wave 1–4 = March 2020–June 2020, July 2020–January 2021, February 2021–June 2021, and July 2021–January 2022; (b) an existing biomarker classifier using IL-6, TNFR1, and bicarbonate levels assigned inflammatory subphenotypes based on a 0.5 probability cutoff [9]. (c) Data are presented for patients included in the biomarker analyses only. Among those who received tocilizumab prior to initiation of mechanical ventilation, the majority received it within two days of starting ventilation. One patient received tocilizumab eight days before mechanical ventilation. (d) Data report the worst observed values of the first 24 h after intubation; (e) data report the values observed 8 h after intubation; between-group comparisons were performed using *t*-tests or Mann–Whitney *U* tests for continuous variables, and Chi-squared tests for categorical variables. *P*-values for categorical data refer to comparisons across all levels

*BMI* body mass index, *COPD* chronic obstructive pulmonary disease, *SOFA* Sequential Organ Failure Assessment Score (without the value for central nervous system)

### Plasma biomarkers over time

**Fig. 1:**
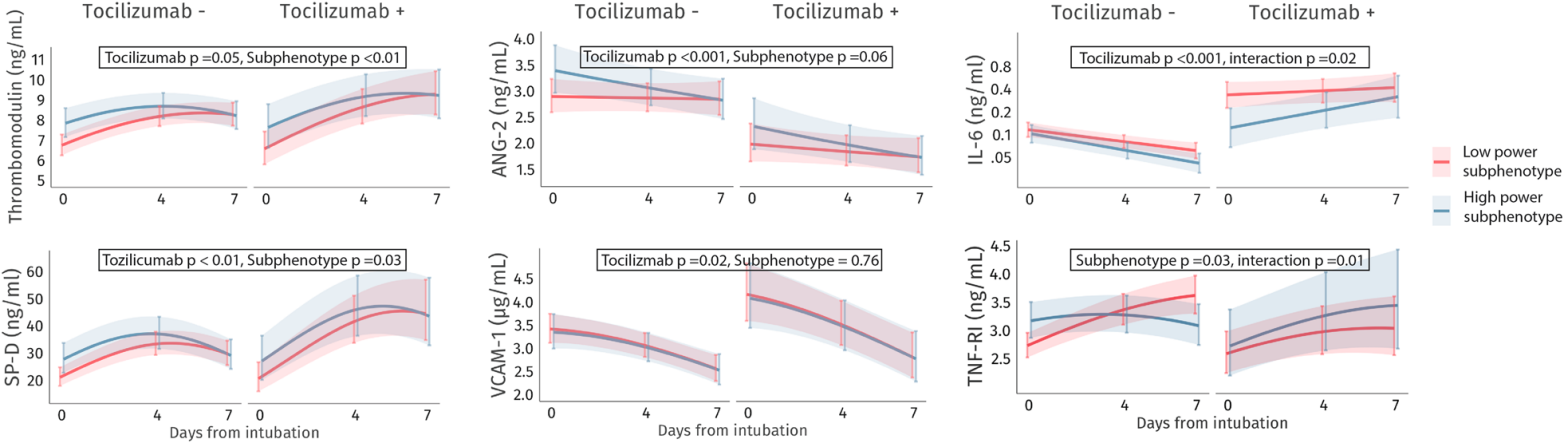
Predictions of biomarker temporal trends. Mean changes in biomarker levels estimated with a generalized linear mixed-effects model for repeated measures. The I bars represent 95% confidence intervals. Low-power subphenotype/no tocilizumab: 209 (48%) of patients; Low-power subphenotype/tocilizumab: 59 (14%); High-power subphenotype/no tocilizumab: 133 (31%); High-power subphenotype/tocilizumab: 26 (6%). *Tocilizumab+* patients treated with tocilizumab, *Tocilizumab−* patients not treated with tocilizumab, *ANG-2* angiopoietin-2, *IL-6* interleukin-6, *SP-D* surfactant protein-D, *TNF-RI* tumor necrosis factor-receptor 1, *VCAM-1* vascular cell adhesion molecule-1

Plasma samples were available for 427 (59%) patients, of which 268 (63%) in the low-power and 159 (37%) in the high-power subphenotype. Concentration differences in biomarkers between subphenotypes were observed at T0 for all biomarkers, except for VCAM-1 (Table S1). After adjustment for tocilizumab treatment, the high-power subphenotype exhibited significantly higher plasma concentrations of SP-D, thrombomodulin and TNF-RI at T0 compared to the low-power subphenotype, but these differences resolved by T4–T7 (Fig. 1, Table S2). Tocilizumab treatment was associated with higher IL-6, SP-D and VCAM-1 and lower angiopoietin-2 levels at T0, trends that persisted over time (Fig. 1, Table S2). Additionally, there was significant interaction between the subphenotypes and tocilizumab (Fig. 1, Table S3). In the high-power subphenotype, tocilizumab treatment was associated with a significantly larger increase in TNF-RI and IL-6 levels over time, compared to the low-power subphenotype.

### Variance explained by tocilizumab and subphenotypes

**Fig. 2:**
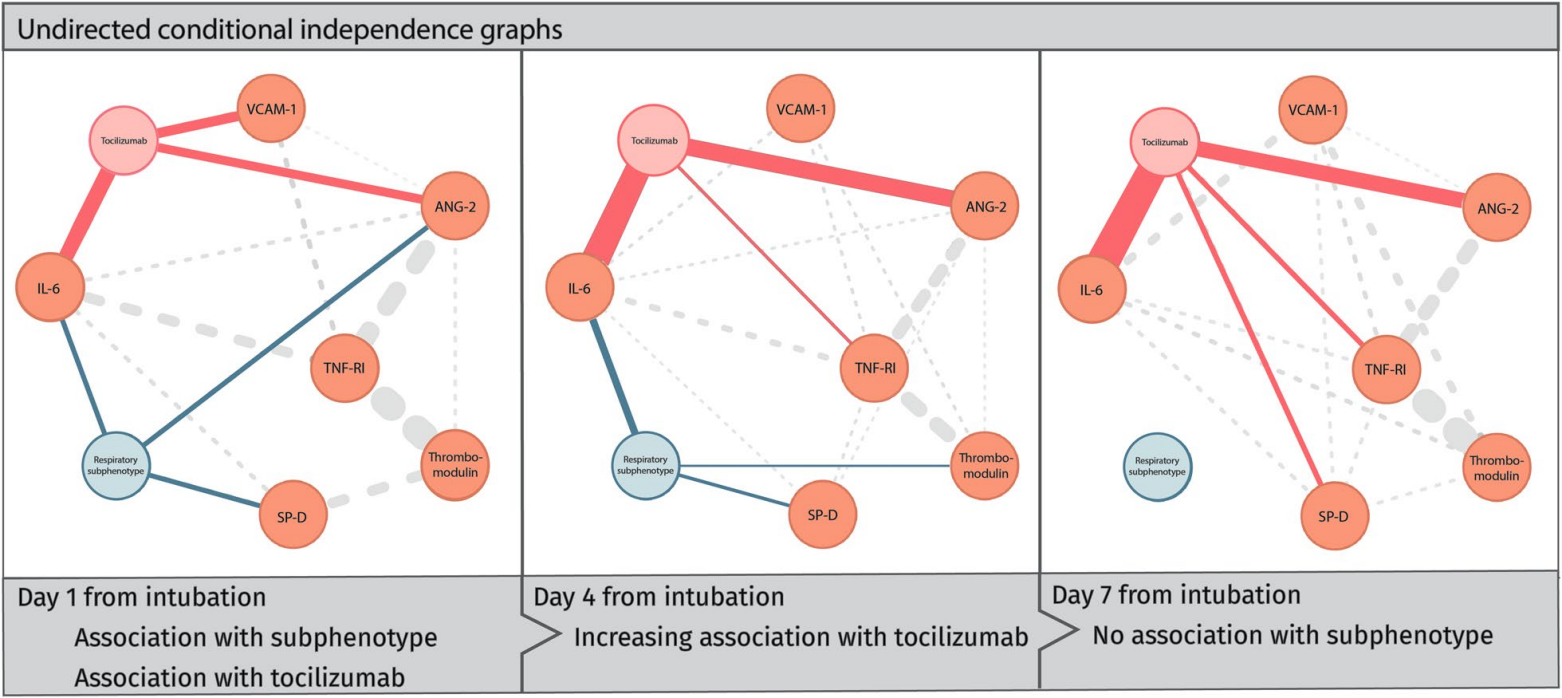
Conditional relationships of subphenotype, tocilizumab treatment and immunobiology. Undirected conditional independence graph of relationships between subphenotype, tocilizumab treatment and biomarkers at T4 and T7, constructed using mixed graphical modeling. The models were fit with using tenfold cross-validation for lambda selection for penalization and included only two-way interactions. The solid edges reflect relationships of biomarkers with either subphenotype (blue) or tocilizumab treatment (red), the dashed edged reflect relationships between biomarkers. The width of the lines reflects the strength of the relationship

Tocilizumab treatment explained approximately four times more of the variance in IL-6 and angiopoietin-2 compared to subphenotype (marginal *R*^2^: 0.145 with treatment and subphenotype vs. 0.026 with subphenotype alone for IL-6, and 0.053 vs. 0.007 for angiopoietin-2, respectively). For biomarkers that significantly differed between low-power and high-power subphenotypes, subphenotype accounted for only a small proportion of the variance and explained only slightly more than tocilizumab use in TNF-RI and thrombomodulin (marginal *R*^2^: 0.023 with subphenotype vs. 0.018 with treatment, and 0.032 vs. 0.027 for thrombomodulin, respectively). Multivariate mixed graphical models confirmed that while there were conditional associations between biomarkers and subphenotype, the associations with tocilizumab use were more evident and persisted over time (Fig. 2).

### Outcomes

At day 90, 110 of 256 (42%) patients with the high-power subphenotype and 151 (32%) patients with the low-power subphenotype died (adj. HR 1.40, 95% CI 1.08–1.82, *p* = 0.012, Table 2, S4). The response to tocilizumab did not differ significantly across subphenotypes (adj. HR 1.18, 95% CI 0.60–2.33, *p* = 0.588, Table 2, Figure S7, Tables S5–6, Figures S5–6).

**Table 2:** Risk estimates for mortality

No. of events/total no. (%)		*P* value
Subphenotypes in relation to outcomes^†^		
90-day mortality		
Low-power subphenotype	151/464 (32)	
High-power subphenotype	110/256 (42)	
Crude analysis—hazard ratio (95%CI)	1.47 (1.15–1.88)	0.002
Adjusted analysis–hazard ratio (95%CI)*	1.40 (1.08–1.82)	0.012
Interaction between subphenotypes and tocilizumab treatment in relation to outcomes^‡^		
90-day mortality		
Crude IPTW analysis—hazard ratio (95%CI)	1.29 (0.66–2.54)	0.454
Adjusted IPTW analysis—hazard ratio (95%CI)*	1.18 (0.60–2.33)	0.588

^†^All models are structured such that a higher odds or hazard ratio represent a poorer outcome for the high-power subphenotype. The analyses included all patients (*n* = 720), using a complete dataset

^‡^All models are structured such that a higher odds or hazard ratio represent less benefit of tocilizumab in the high-power subphenotype, compared to the low-power subphenotype. The analyses include a weighted pseudopopulation, using a complete dataset

^*^The analyses included adjustment for age, sex, BMI, admission FiO_2_, admission Sequential Organ Failure Score (minus central nervous system score), admission C-reactive protein, site and time period

## Discussion

In this study, we assessed respiratory trajectories and tocilizumab treatment as predictors of immunobiology and mortality in patients with COVID-19-related ARDS. We observed differences in immunobiological patterns across subphenotypes, along with differential TNF-RI and IL-6 responses following tocilizumab administration. However, these subphenotype-associated differences were minor compared to the overall immunobiological response to tocilizumab.

Tocilizumab, rather than subphenotype, accounted for most of the variance in biomarker trajectories. Its effects were largely consistent across subphenotypes, with differences observed only in the inflammatory biomarkers IL-6 and TNF-RI, neither of which led to substantial differences in overall plasma concentrations. In fact, TNF-RI levels were more similar between subphenotypes in tocilizumab-treated patients than in those not treated, and the effect of tocilizumab on IL-6 levels far outweighed any subphenotype-related differences. Notably, the association between tocilizumab and 90-day mortality likewise did not differ significantly by subphenotype.

Although the variance explained by either tocilizumab or subphenotype was modest, the observed immunobiological patterns were consistent with known mechanisms. Previous studies have shown increases in IL-6 plasma concentrations after tocilizumab, likely due to compensatory upregulation of IL-6 production in response to IL-6 receptor blockade [10–12]. A rise in thrombomodulin over time after tocilizumab has also been reported [10]. The immunobiological differences between the subphenotypes reflected their physiological characteristics. The high-power subphenotype, marked by increasing mechanical power and ventilatory ratio, exhibited significantly higher levels of SP-D, TNF-RI and thrombomodulin, and a trend towards higher angiopoietin-2. Elevated SP-D—produced by alveolar type II cells—indicates epithelial damage [13, 14], which can impair surfactant production, increase alveolar surface tension, lower compliance, and reduce edema clearance [2, 15, 16]. Similarly, higher levels of angiopoietin-2 and thrombomodulin suggest endothelial damage [17]. Endothelial injury promotes a procoagulant state [18], which may contribute to the increased ventilatory ratios observed in the high-power subphenotype.

Previous evidence highlights the challenges of subphenotype-targeted immunomodulation. For instance, secondary analyses of randomized controlled trials found simvastatin to be effective only in ARDS patients with a hyperinflammatory subphenotype [7, 19, 20]. However, simvastatin was effective in critically ill COVID-19 patients, despite 98.8% of these patients belonging to the hypoinflammatory subphenotype [21]. This suggests that the immunobiological response to simvastatin is more complex than can be explained by the hypo- and hyperinflammatory subphenotype classification. In our predominantly hypoinflammatory study population, we observed two subphenotypes distinguished not by inflammation, but by markers of endothelial/epithelial damage and immunothrombosis. The overlap among various identifiable subphenotypes is an area of great interest, highlighting the need to better understand their shared biological traits, often referred to as treatable traits [4]. The overall dominant effect of tocilizumab, largely unaffected by subphenotype, argue against the use of respiratory subphenotypes as a basis for initiating or withholding tocilizumab. Future studies should assess whether other therapeutic strategies targeting the underlying pathophysiological pathways of endothelial and epithelial damage or immunothrombosis offer differential benefits across respiratory subphenotypes.

Our study has several limitations. First, the non-randomized allocation of tocilizumab may have introduced bias, as during the study period clinical practice evolved from using no immunomodulation to widespread use of corticosteroids and tocilizumab. Moreover, tocilizumab—administered at or near ICU admission—may affect subphenotype allocation. This extended inclusion period also introduced potential bias from changing COVID-19 variants, vaccination statuses, and treatment strategies beyond immunomodulation. Second, our study may have been underpowered to fully assess all relationships, with limited biomarker data available in some subgroups. Third, our study included exclusively COVID-19-related ARDS. While we believe these findings may extend to ARDS in general, generalizations beyond COVID-19-related ARDS should be made with caution. Lastly, ventilation data of the first 4 days are needed to classify patients which limits the clinical usability. However, recent studies have created an accurate prediction model using data of only the first 2 days [22].

This study has several strengths. First, our use of longitudinal data allowed us to capture immunobiological changes over time, providing a dynamic perspective on the relationship between subphenotypes, tocilizumab response, and key inflammatory pathways. Second, we conducted a detailed biomarker analysis, incorporating multiple markers of epithelial and endothelial injury, which enhances our understanding of the distinct biological mechanisms underlying respiratory subphenotypes. Third, the study benefits from a large sample size, increasing statistical power and the reliability of our findings. Lastly, we focused on a homogeneous disease population—patients with COVID-19-related ARDS—which minimizes variability and ensures that observed differences are driven by biological mechanisms rather than heterogeneous causes of ARDS.

## Conclusion

Our study provides insight into how temporal immunobiology in COVID-19-related ARDS differs between respiratory trajectory subphenotypes. The high-power subphenotype exhibited higher levels of markers of endothelial and epithelial damage and immunothrombosis at intubation, which may partially explain its progression toward higher mechanical power and ventilatory ratio and higher mortality. However, compared to the overwhelming immunobiological response to tocilizumab, these between-group differences were only minor, suggesting that respiratory subphenotype-based immunomodulation may not be justified.

## Supplementary Information

Supplementary Material 1.

## Data Availability

The datasets used during the current study are available from the corresponding author on reasonable request.
