# Supplementary material for: Immunobiological effects of tocilizumab across respiratory subphenotypes in COVID-19 ARDS
Source: Intensive Care Med Exp. 2025 Jul 9;13:70. doi: 10.1186/s40635-025-00779-z (PMC12240919; doi:10.1186/s40635-025-00779-z)
Supplement: Supplementary file 1 — Supplementary Material 1. [file 40635_2025_779_MOESM1_ESM.docx]

Online supplementary material to:

**Respiratory trajectory subphenotypes and immunomodulation in COVID-19 related ARDS**

Inhoud

[1. Additional methods: 3](#_Toc193349792)

[1.1 Data extraction and pre-processing 3](#_Toc193349793)

[1.2 Calculation of respiratory variables 4](#_Toc193349794)

[1.3 Quality control of biomarker measurements 4](#_Toc193349795)

[1.3.1 Quality control of biomarker measurements conducted 5](#_Toc193349796)

[1.3.2 Plates and sample types 5](#_Toc193349797)

[1.3.3 Average intra- and inter-Assay Coefficient of Variance (CV%) 5](#_Toc193349798)

[1.3.4 Summary of sample detection results (UMC Utrecht) 6](#_Toc193349799)

[1.4 Statistical analysis 6](#_Toc193349800)

[1.4.2 Imputation of missing data 6](#_Toc193349801)

[1.4.3 Inverse probability of treatment weighting 7](#_Toc193349802)

[2. Supplementary tables: 8](#_Toc193349803)

[Table S1. Biomarker concentrations 8](#_Toc193349804)

[Table S2. Relation between subphenotype, immunomodulation and biomarker temporal trends 9](#_Toc193349805)

[Table S3. Relation between subphenotype, immunomodulation and biomarker temporal trends, including interaction terms 10](#_Toc193349806)

[Table S4. Cox proportional hazards analysis of the relation between subphenotype and 90-day mortality 11](#_Toc193349807)

[Table S5. Odds ratios (95% CI) of receiving tocilizumab, for all variables included in the propensity score model. 12](#_Toc193349808)

[Table S6. Inverse probability of treatment weighted survival analysis of the relation between tocilizumab and 90-day mortality across subphenotypes 13](#_Toc193349809)

[Table S7A. Missing clinical and biomarker data 14](#_Toc193349810)

[Table S7B. Missing respiratory data 15](#_Toc193349811)

[3. Supplementary figures: 16](#_Toc193349812)

[Figure S1. Flowchart of data reshaping from export to dataset. 16](#_Toc193349813)

[Figure S2. Flowchart of determination of respiratory status at each 8hr time interval. 17](#_Toc193349814)

[Figure S3. Patient inclusion flowchart 18](#_Toc193349815)

[Figure S4. Profile plot of classified subphenotypes 19](#_Toc193349816)

[Figure S5. Distribution of propensity scores 20](#_Toc193349817)

[Figure S6. Standardized mean differences before and after IPTW 21](#_Toc193349818)

# 1. Additional methods:

## 1.1 Data extraction and pre-processing

We extracted electronic health record (EHR) data from Epic (Epic Systems Corporation, Verona, United States) for AUMC patients and from MetaVision (iMDsoft, Tel Aviv, Israel) for UMCU patients. Data was extracted for patients admitted to the intensive care unit (ICU) between February 1, 2020, and May 31, 2022, with either a COVID-19 admission diagnosis code or a pneumonia admission diagnosis code and manual labeling as COVID-19 (based on PCR results). The admission reasons of the included patients were manually validated by the study team. The data was pre-processed and mapped to an analyzable dataset separately for each participating ICU before being merged. The extracted data included demographics, clinical disease severity scores, laboratory measurements, data from vital sign monitors and mechanical ventilators and administered medication. Extracted data covered the entire hospital and ICU stay per patient. Complete data on mortality from the Dutch national registry (Centraal Bureau van de Statistiek) are linked to EHR systems every four months, and these data were used in this study.

Data pre-processing included cleaning (e.g. removal of impossible values), computing a variable that indicated if a patient had received mechanical ventilation, and reducing the highly granular (minute to hourly) data to the 8-hourly respiratory data necessary to predict subphenotype membership. Patients were labelled as having mechanical ventilation based on:

1. the availability of tidal OR minute volumes;
   AND
2. a mechanical ventilator mode indicating invasive mechanical ventilation.

The first timepoint where this occurred was considered the timepoint of inclusion in the study. Baseline respiratory variables were selected 1 hour after this point. From this point forward, 12 timepoints at 8-hour intervals were created up to 96 hours post-inclusion. At each of these timepoints, the nearest available arterial blood gas (ABG) was selected and the closest available respiratory values before the ABG were taken. See figure E1 for a specification of the collected data.

## 1.2 Calculation of respiratory variables

| Name | Abbreviation | Formula | Units |  |
| --- | --- | --- | --- | --- |
| CO_2_ difference | CO_2_ diff | $\mathrm{PaCO}_{2} - \mathrm{etCO}_{2}$ | mmHg |  |
| Driving Pressure (dynamic) | ΔP | $P_{max}- PEEP$ | cmH_2_O |  |
| Compliance (dynamic) | Crs | $\frac{V_{T}}{Driving pressure}$ | mL/cmH_2_O |  |
| Fractional dead space | - | $\frac{{PaCO}_{2} - \mathrm{etCO}_{2}}{{PaCO}_{2}}$ | Index |  |
| Minute volume | MinVol | $V_{T} \times RR$ | L/min |  |
| Mechanical Power | MP | $0.098 \times V_{T} \times RR \times(Pmax-Pdriving \div2)$  Where RR and Pmax are assessed during pressure-controlled ventilation. | J/min |  |
| PaO_2_ / FiO_2_ | - | $\frac{{PaO}_{2}}{{FiO}_{2}}$ | mmHg |  |
| pH | - | - | Index |  |
| Ventilatory Ratio | VR | ${MinVol}_{m} \times{PaCO}_{2, m} \div{MinVol}_{p} \times{PaCO}_{2, p}$  Where m is measured and p is predicted. | Index |  |
| Abbreviations: PaCO_2_, partial pressure of carbon dioxide; et–CO_2_, end–tidal carbon dioxide; Pmax, maximum airway pressure; PEEP, positive end–expiratory pressure; V_T_, tidal volume; RR, respiratory rate; PaO_2_, partial pressure of oxygen; FiO_2_, fraction of inspired oxygen. Compliance, mechanical power and driving pressure are only calculated in patients that received controlled ventilation. | | | | |

## 1.3 Quality control of biomarker measurements

Biomarkers were measured in separate batches for the Amsterdam University Medical Centers (AUMC) and the University Medical Center Utrecht (UMCU).

### 1.3.1 Quality control of biomarker measurements conducted

Biomarker measurements were performed by Arcadia, UMCU, the Netherlands. Samples were randomized across plates. To ensure the quality of individual temporal trends, all (three) samples per individual were placed on a single plate. Study samples were measured in one-fold. Eight standards were included on every plate in duplicate, to generate five-parameter logistic curves.

Intra- and inter assay coefficients of variances (CVs) were based on internal control samples: six EDTA- and four lithium-heparin plasma study samples, and one pooled plasma sample. Calculations were made using the Observed concentration values within the limits of quantification.

Principal component analysis (PCA) was used to assess if there were signs of plate or batch effects. None of the 15 plates used yielded substantially different measured values, when values were visualized in PCA plots. Thus, we did not correct for plate effects.

### 1.3.2 Plates and sample types

Both ethylenediaminetetraacetic acid (EDTA)- and lithium-heparin plasma samples were utilized in the study (760 and 91, respectively). When both types were available from the same blood draw, preference was given to EDTA plasma for analysis. To evaluate the consistency of measurements across these plasma types, one plate included 32 paired samples of both EDTA and lithium-heparin plasma from the same blood draws. The measurements demonstrated a near-perfect correlation (Pearson r > 0.98) between the two plasma types, with negligible systematic differences in absolute concentration.

### 1.3.3 Average intra- and inter-Assay Coefficient of Variance (CV%)

|  | Angiopoietin-2 | IL-6 | SP-D | Thrombomodulin | TNF-RI | VCAM-1 |
| --- | --- | --- | --- | --- | --- | --- |
| Average Intra-CV%^1^ | 2.6 | 2.0 | 2.6 | 2.2 | 2.6 | 3.6 |
| Average Inter-CV%^1^ | 8.3 | 11.9 | 14.1 | 6.6 | 8.0 | 12.0 |
| ^1^Values were calculated using the Observed concentration of the values between the lower- and upper limit of quantification. | | | | | | |

### 1.3.4 Summary of sample detection results (UMC Utrecht)

|  | Number (%) of samples with values that could be detected or extrapolated^1^ | Upper- and lower limit of quantification^2^ (pg/ml) |
| --- | --- | --- |
| Angiopoietin-2 | 850 (100) | 13.9 – 41780 |
| IL-6 | 838 (99) | 0.8 – 2380 |
| SP-D | 811 (95) | 32.9 – 96308 |
| Thrombomodulin | 850 (100) | 8.6 – 25072 |
| TNF-RI | 850 (100) | 17 – 49579 |
| VCAM-1 | 840 (99) | 1527 – 4506800 |
| ^1^Values that could not be extrapolated on the 5-PL curve, were replaced with 0.5*LLOQ or with the maximum of the extrapolated values for the analyses.  ^2^Median LLOQ and ULOQ of fifteen measured plates. | | |

## 1.4 Statistical analysis

### 1.4.2 Imputation of missing data

We used a two-step multiple imputation by chained equations (MICE) process to handle missing data. In the first step, respiratory data was imputed separately for each site, to account for dependency in the data due to differences in mechanical ventilation strategies. These MICE models included all available (i.e. 8-hourly) respiratory data and baseline characteristics. From each site, one imputed dataset was selected and combined with data from other sites for allocation to respiratory trajectory subphenotypes, as pooling longitudinal LCA across multiple imputed datasets was not feasible. In the second step, missing covariate data for outcome analyses was imputed across all sites simultaneously. This imputation included subphenotype allocation as a covariate but excluded 8-hourly respiratory data. For both steps, predictive mean matching was used and density- and convergence plots were assessed to confirm that the imputed data closely matched the original data distribution.

As a sensitivity analysis for step 1, we employed a more complex imputation strategy to account for longitudinal dependencies in the data. The prediction matrix was adjusted such that, for each variable at each time point, all cross-sectional data and baseline characteristics were used along with data from all other time points of the same variable (e.g., predicting mechanical power at time point 8 included baseline characteristics, other respiratory variables at time point 8, and mechanical power at time points 1-7 and 9-12). This approach yielded respiratory subphenotype allocations similar to the primary method.

### 1.4.3 Inverse probability of treatment weighting

*Rationale*We used inverse probability of treatment weighting (IPTW) based on propensity scores (PS) to reduce baseline imbalances between patients treated with tocilizumab and those not treated. PS methods were preferred over direct covariate adjustment (or adjustment with PS) because they allow for assessment of balance before the final analysis. IPTW was chosen over other PS methods (i.e. matching) to retain the entire study sample, preserving generalizability.

*Estimation of propensity scores*We fitted a logistic regression model of tocilizumab regressed on baseline covariates, to obtain the predicted probability of receiving tocilizumab. Covariates were selected based on their effect on the relation between exposure and outcome, or their relation with outcome. Covariates that were only related to exposure were not included [Brookhart Am J Epidemiology 2006]. A single logistic regression model was used to estimate PS for all patients, instead of per subphenotype, as the risk factors for receiving treatment were considered consistent across subphenotypes.

# 2. Supplementary tables:

## Table S1. Biomarker concentrations

| Biomarker levels (pg/mL) (median/IQR) | Subphenotype 1 (n=268) | Subphenotype 2 (n=159) | p-value |  |
| --- | --- | --- | --- | --- |
| Angiopoietin-2  T0  T4  T7 | 2619 [1497 – 4388] (n=242)  2313 [1381 – 4032] (n=243)  2219 [1409 – 4312] (n=213) | 2951 [1675 – 5350] (n=146)  2762 [1565 – 4917] (n=150)  2543 [1537 – 4666] (n=134) | 0.048  0.123  0.285 |  |
| Interleukin-6  T0  T4  T7 | 130 [44 - 444] (n= 236)  126 [31 – 426] (n=239)  98 [22 – 320] (n=208) | 98 [42 - 235] (n=142)  80 [21 – 233] (n=145)  57 [23 – 158] (n=131) | 0.070  0.020  0.036 |  |
| Surfactant protein-D  T0  T4  T7 | 24016 [10081 – 49249] (n=228)  39001 [21951 – 61355] (n=238)  39193 [20476 – 66932] (n=215) | 33370 [13235 – 61128] (n=137)  36979 [20787 – 62872] (n=141)  32683 [18391 – 62819] (n=131) | 0.027  0.645  0.304 |  |
| Thrombomodulin  T0  T4  T7 | 6816 [5058 – 9329] (n=246)  8265 [6179 – 11291] (n=250)  8383 [6127 – 11063] (n=226) | 7569 [5904 – 10009] (n=150)  8802 [6695 – 11453] (n=151)  8778 [6256 – 11153] (n=141) | 0.005  0.237  0.642 |  |
| TNF-receptor 1  T0  T4  T7 | 2477 [1913 – 3450] (n=228)  2886 [2062 – 4450] (n=238)  3183 [2309 – 4645] (n=215) | 2834 [2203 – 4159] (n=138)  3194 [2289 – 4398] (n=141)  3170 [2240 – 4565] (n=130) | 0.008  0.314  0.443 |  |
| VCAM-1  T0  T4  T7 | 3704550 [2401200 - 5777025] (n=236)  3184500 [2056900 – 4911600] (n=239)  2777050 [1933500 – 4242000] (n=208) | 3646300 [2251525 – 5398175] (n=142)  3154000 [2211600 – 4609200] (n=145)  28090800 [1934800 - 4038700] (n=131) | 0.520  0.903  0.690 |  |

Biomarker levels were compared between subphenotypes using Mann-Whitney U tests. T0 is the day of intubation, T4 is day 4 after intubation, and T7 is day 7 after intubation. If a sample was not available on the specified day, a sample of either the day before or after was used. Measurements were conducted using leftover EDTA- or heparin plasma samples. TNF-receptor 1: tumor necrosis factor receptor 1; VCAM-1: Vascular cell adhesion molecule-1.

|  | *Angiopoietin-2* | *Interleukin-6* | *SP-D* | *Thrombomodulin* | *TNF-R1* | *VCAM-1* |
| --- | --- | --- | --- | --- | --- | --- |
| *Intercept* | | | | | | |
| *β*  *p-value* | 7.97  <0.001* | 4.82  <0.001* | 9.96 <0.001* | 8.82  <0.001* | 7.92  <0.001* | 15.0  <0.001* |
| *Time* | | | | | | |
| *β^2^*  *p-value*  *β^2^*  *p-value* | -0.00  0.782  -  - | -0.10  <0.001*  -  - | 0.20  <0.001*  -0.02  <0.001* | 0.07  <0.001*  -0.01  <0.001* | 0.06  <0.001*  -0.00  0.014* | -0.01  0.519  -0.00  0.028* |
| *Subphenotype* | | | | | | |
| *β*  *p-value* | 0.16  0.061 | -0.30  0.066 | 0.27  0.025* | 0.15  0.008* | 0.13  0.028* | -0.02  0.762 |
| *Immunomodulation* | | | | | | |
| *Tocilizumab - β*  *p-value* | -0.38  <0.001* | 0.77  <0.001* | -0.02  0.872 | -0.03  0.692 | -0.09  0.209 | 0.20  0.016* |
| *Time*Subphenotype* | | | | | | |
| *β*  *p-value* | -0.02  0.071 | -0.01  0.719 | -0.04  0.029* | -0.02  0.009* | -0.03  <0.001* | 0.00  0.934 |
| *Time*Immunomodulation* | | | | | | |
| *Tocilizumab - β*  *p-value* | -0.02  0.303 | 0.17  <0.001* | 0.06  0.008* | 0.02  0.052 | 0.00  0.927 | -0.01  0.272 |

## Table S2. Relation between subphenotype, immunomodulation and biomarker temporal trends

Mixed-effects models estimate log(ng/ml). All models used ‘subphenotype 1’ and ‘no tocilizumab’ as the reference category. A polynomial term for time was only included if this significantly improved model fit (based on Akaike’s Information Criterion and likelihood ratio tests). Beta-coefficients were obtained using restricted maximum likelihood. SP-D: surfactant protein-D. TNF-R1: tumor necrosis factor receptor 1; VCAM-1: vascular cell adhesion molecule 1.

## Table S3. Relation between subphenotype, immunomodulation and biomarker temporal trends, including interaction terms

|  | *Angiopoietin-2* | *Interleukin-6* | *SP-D* | *Thrombomodulin* | *TNF-R1* | *VCAM-1* |
| --- | --- | --- | --- | --- | --- | --- |
| *Intercept* | | | | | | |
| *β*  *p-value* | 7.96  <0.001* | 4.75  <0.001* | 9.96 <0.001* | 8.82  <0.001* | 7.91  <0.001* | 15.1  <0.001* |
| *Time* | | | | | | |
| *β^2^*  *p-value*  *β^2^*  *p-value* | -0.00  0.755  -  - | -0.09  <0.001*  -  - | 0.20  <0.001*  -0.02  <0.001* | 0.08  <0.001*  -0.01  <0.001* | 0.07  <0.001*  -0.00  0.013* | -0.01  0.532  -0.00  0.028* |
| *Subphenotype* | | | | | | |
| *β*  *p-value* | 0.18  0.058 | -0.13  0.483 | 0.27  0.044* | 0.15  0.016* | 0.15  0.022* | -0.04  0.575 |
| *Immunomodulation* | | | | | | |
| *Tocilizumab - β*  *p-value* | -0.35  0.005* | 1.06  <0.001* | -0.02  0.901 | -0.03  0.762 | -0.05  0.524 | 0.16  0.110 |
| *Time*Subphenotype* | | | | | | |
| *β*  *p-value* | -0.02  0.123 | -0.04  0.177 | -0.04  0.084 | -0.03  0.005* | -0.04  <0.001* | 0.00  0.971 |
| *Time*Immunomodulation* | | | | | | |
| *Tocilizumab - β*  *p-value* | -0.01  0.460 | 0.12  <0.001* | 0.07  0.013* | 0.01  0.335 | -0.02  0.178 | -0.02  0.343 |
| *Subphenotype*Immunomodulation* | | | | | | |
| *Tocilizumab - β*  *p-value* | -0.11  0.625 | -0.88  0.033 | -0.01  0.986 | -0.00  0.980 | -0.10  0.501 | 0.11  0.507 |
| *Time*Subphenotype*Immunomodulation* | | | | | | |
| *Subphen2*Tocilizumab - β*  *p-value* | -0.006  0.867 | 0.14  0.022* | -0.02  0.617 | 0.02  0.280 | 0.06  0.014* | 0.00  0.930 |

Mixed-effects models estimate log(ng/ml). All models used subphenotype 1 and ‘no tocilizumab’ as the reference category. A polynomial term for time was only included if this significantly improved model fit (based on Akaike’s Information Criterion and likelihood ratio tests). Beta-coefficients were obtained using restricted maximum likelihood. TNF-receptor 1: tumor necrosis factor receptor 1; VCAM-1: vascular cell adhesion molecule 1.

## Table S4. Cox proportional hazards analysis of the relation between subphenotype and 90-day mortality

| N=720 | Hazard ratio (95% CI) | p-value |
| --- | --- | --- |
| Subphenotype | 1.40 (1.08 – 1.82) | 0.012* |
| Demographics  Age  Sex  Body mass index | 1.06 (1.04 – 1.07)  1.24 (0.92 – 1.66)  0.98 (0.95 – 1.00) | <0.0001*  0.159  0.076 |
| Vital status at ICU presentation  SOFA  FiO2 | 1.08 (1.02 – 1.14)  1.01 (1.00 – 1.01) | 0.008*  0.061 |
| Laboratory values at ICU presentation  C-reactive protein | 1.00 (1.00 – 1.00) | 0.427 |
| ICU presentation  Site AUMC – location VUmc  Site UMC Utrecht  Time period | 0.38 (0.32 – 0.57)  0.43 (0.27 – 0.52)  1.00 (1.00 – 1.00) | <0.0001*  <0.0001*  0.272 |

Time period was computed as a numerical value per calendar day, with patients admitted at a later calendar date having a higher number. ICU: intensive care unit; SOFA: Sequential organ failure assessment score (minus central nervous system score).

## Table S5. Odds ratios (95% CI) of receiving tocilizumab, for all variables included in the propensity score model.

| N=720 | OR (95% CI) | p-value |
| --- | --- | --- |
| Intercept | 0.064 (0.008 – 0.485) | 0.009* |
| Age  Sex  Body mass index | 1.003 (0.984 – 1.023)  1.070 (0.697 – 1.658)  1.005 (0.968 – 1.041) | 0.751  0.761  0.805 |
| Medical history  Chronic kidney disease  Chronic lung disease  Diabetes  Immunodeficiency  Cancer | 2.287 (0.980 – 5.196)  1.321 (0.699 – 2.509)  1.156 (0.721 – 1.831)  0.887 (0.452 – 1.689)  1.451 (0.642 – 3.152 ) | 0.051  0.406  0.542  0.721  0.356 |
| Disease severity at ICU presentation  SOFA  FiO2  Vasopression | 0.871 (0.763 – 0.989)  1.014 (1.001 – 1.027)  2.007 (1.087 – 3.787) | 0.037*  0.036*  0.028* |
| Laboratory values at ICU presentation  Leukocyte count  C-reactive protein | 0.982 (0.952 – 1.004)  1.002 (1.000 – 1.005) | 0.209  0.015* |
| ICU presentation  Site AUMC – location VUmc  Site UMC Utrecht  Time period | 1.610 (0.901 – 2.918)  1.937 (1.091 – 3.504)  1.004 (1.003 – 1.006) | 0.111  0.026  <0.0001* |

The model was structured such that a higher odds ratio presents a higher risk of receiving tocilizumab. Time period was computed as a numerical value per calendar day, with patients admitted at a later calendar date having a higher number. ICU: intensive care unit; SOFA: Sequential organ failure assessment score (minus central nervous system score).

## Table S6. Inverse probability of treatment weighted survival analysis of the relation between tocilizumab and 90-day mortality across subphenotypes

| N=720 | Hazard ratio (95% CI) | p-value |
| --- | --- | --- |
| Subphenotype  Tocilizumab Subphenotype*Tocilizumab‡ | 1.37 (1.00 – 1.87)  0.83 (0.55 – 1.26)  1.18 (0.60 – 2.33) | 0.049*  0.384  0.634 |
| Demographics  Age  Sex  Body mass index | 1.06 (1.04 – 1.08)  1.31 (0.90 – 1.90)  0.98 (0.94 – 1.01) | <0.0001*  0.161  0.162 |
| Disease severity at ICU presentation  SOFA  FiO2 | 1.09 (1.01 – 1.17)  1.01 (1.00 – 1.02) | 0.035*  0.053 |
| Laboratory values at ICU presentation  C-reactive protein | 1.00 (1.00 – 1.00) | 0.584 |
| ICU presentation  Site AUMC – location VUmc  Site UMC Utrecht  Time period | 0.30 (0.20 – 0.43)  0.39 (0.27 – 0.56)  1.00 (1.00 – 1.00) | <0.0001*  <0.0001*  0.421 |

‡The model was structured such that a higher odds ratio represents less benefit of tocilizumab in respiratory trajectory subphenotype 2, compared to subphenotype 1. Time period was computed as a numerical value per calendar day, with patients admitted at a later calendar date having a higher number. ICU: intensive care unit; SOFA: Sequential organ failure assessment score (minus central nervous system score).

## Table S7A. Missing clinical and biomarker data

| No. (%) | AUMC – location AMC (n = 221) | AUMC – location VUmc (n=218) | UMCU  (n=281) |
| --- | --- | --- | --- |
| Age  Sex  BMI | 0 (0)  0 (0)  6 (3) | 0 (0)  0 (0)  6 (3) | 0 (0)  0 (0)  0 (0) |
| Medical history  Cancer  Chronic kidney disease  Chronic respiratory failure  Diabetes  Heart failure  Immunodeficiency  Obstructive lung disease | 0 (0)  0 (0)  0 (0)  0 (0)  0 (0)  0 (0)  0 (0) | 0 (0)  0 (0)  0 (0)  0 (0)  0 (0)  0 (0)  0 (0) | 0 (0)  0 (0)  0 (0)  0 (0)  0 (0)  0 (0)  (0) |
| Laboratory values at ICU presentation  Bicarbonate  C-reactive protein  Leukocyte count | 0 (0)  26 (12)  1 (0) | 0 (0)  6 (3)  8 (4) | 0 (0)  2 (1)  2 (1) |
| Vital status at ICU presentation  FiO2  SOFA  Vasopression | 1 (0)  0 (0)  0 (0) | 0 (0)  0 (0)  0 (0) | 0 (0)  0 (0)  0 (0) |
| Biomarker measurements  Angiopoietin-2 (T0)  Angiopoietin-2 (T4)  Angiopoietin-2 (T7)  IL-6 (T0)  IL-6 (T4)  IL-6 (T7)  Thrombomodulin (T0)  Thrombomodulin (T4)  Thrombomodulin (T7)  TNF-RI (T0)  TNF-RI (T4)  TNF-RI (T7)  SP-D (T0)  SP-D (T4)  SP-D (T7)  VCAM-1 (T0)  VCAM-1 (T4)  VCAM-1 (T7) | 124 (56)  127 (58)  164 (74)  135 (61)  136 (62)  174 (79)  118 (53)  119 (54)  146 (66)  148 (67)  141 (64)  146 (76)  149 (67)  141 (64)  167 (76)  135 (61)  136 (62)  174 (79) | 193 (89)  192 (88)  195 (89)  192 (88)  192 (88)  193 (89)  191 (88)  192 (88)  193 (89)  191 (88)  192 (88)  194 (89)  191 (88)  192 (88)  193 (89)  192 (88)  192 (88)  193 (89) | 15 (5)  8 (3)  14 (5)  15 (5)  8 (3)  14 (5)  15 (5)  8 (3)  14 (5)  15 (5)  8 (3)  14 (5)  15 (5)  8 (3)  14 (5)  15 (5)  8 (3)  14 (5) |
| Outcome data  ICU mortality  Survival until day 90 | 0 (0)  0 (0) | 0 (0)  0 (0) | 0 (0)  0 (0) |

ICU: intensive care unit; SOFA: Sequential organ failure assessment score (minus central nervous system score); IL-6: Interleukin-6, TNF-RI: Tumor necrosis factor receptor-1; SP-D: Surfactant protein-D; VCAM-1: Vascular cell adhesion molecule-1.

## Table S7B. Missing respiratory data

| No. (%) | AUMC – location AMC (n= 2873) | AUMC – location VUmc (n=2834) | UMCU  (n= 3653) |
| --- | --- | --- | --- |
| CO2-difference | 75 (3) | 29 (1) | 415 (12) |
| Compliance | 102 (4) | 52 (2) | 88 (2) |
| Driving Pressure | 33 (1) | 15 (1) | 86 (2) |
| Minute Volume | 27 (1) | 15 (1) | 84 (2) |
| Mechanical Power | 28 (1) | 16 (1) | 88 (2) |
| pH | 66 (2) | 23 (1) | 393 (11) |
| PF-ratio | 78 (3) | 26 (1) | 441 (12) |
| Ventilatory ratio | 96 (3) | 99 (3) | 393 (11) |

# 3. Supplementary figures:

## Figure S1. Flowchart of data reshaping from export to dataset.


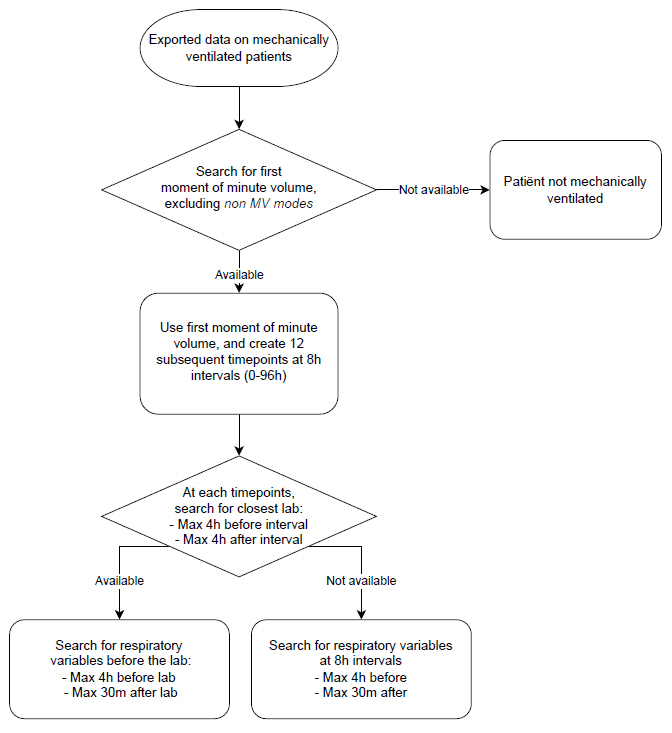


Abbreviations: MV = mechanical ventilation.

## Figure S2. Flowchart of determination of respiratory status at each 8hr time interval.


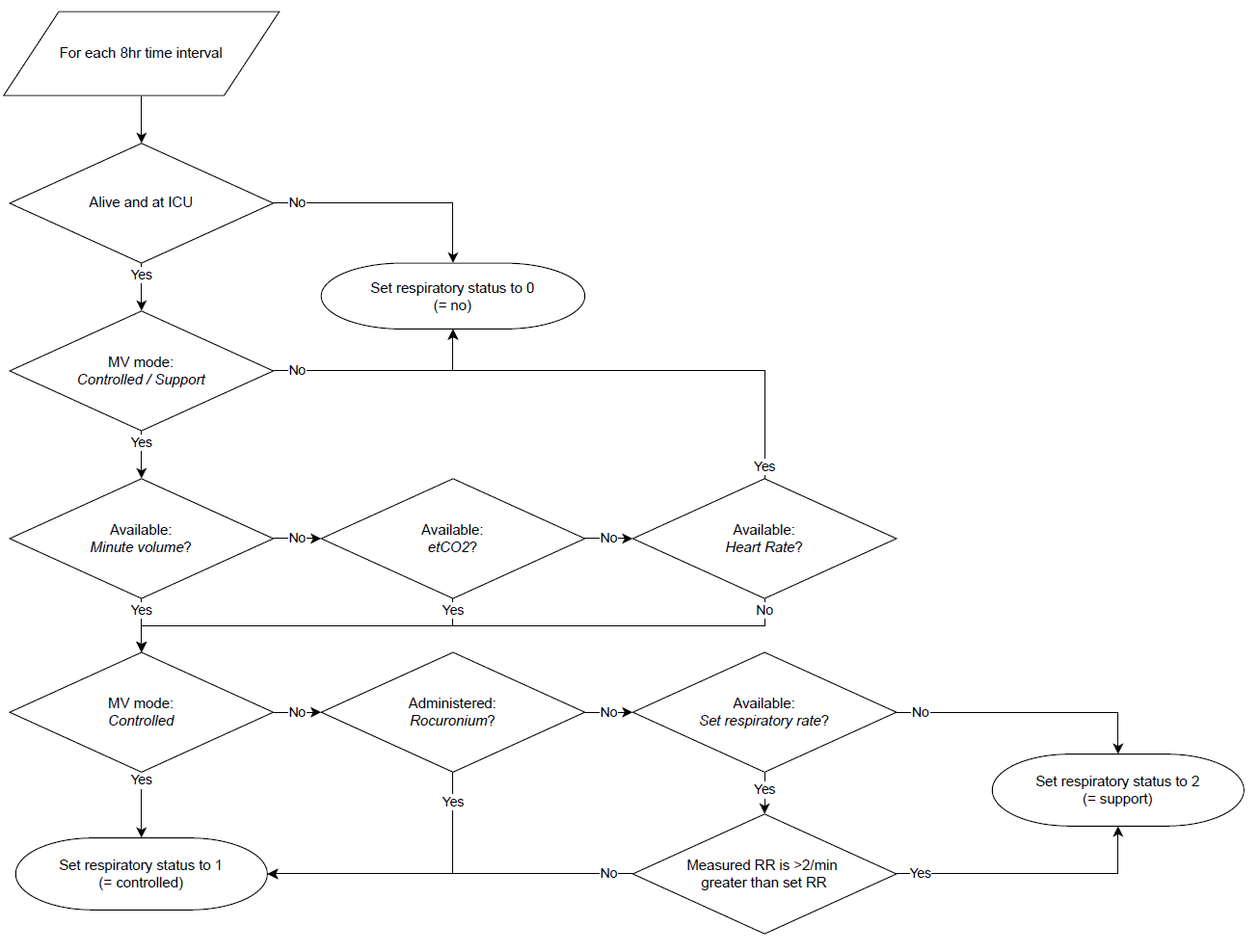


Abbreviations: ICU = intensive care unit, MV = mechanical ventilation, RR = respiratory rate.

## Figure S3. Patient inclusion flowchart

Abbreviations: AUMC = Amsterdam UMC, UMCU = UMC Utrecht, MV = Mechanical ventilation, COVID-19 = Coronavirus disease 2019, ECMO = Extracorporeal membrane oxygenation.

**
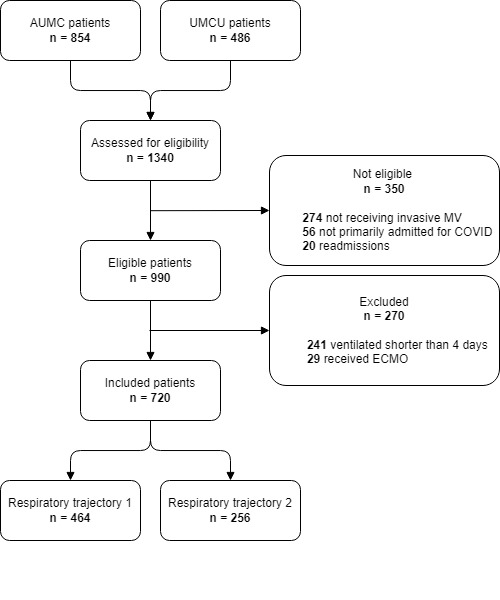
**

## Figure S4. Profile plot of classified subphenotypes


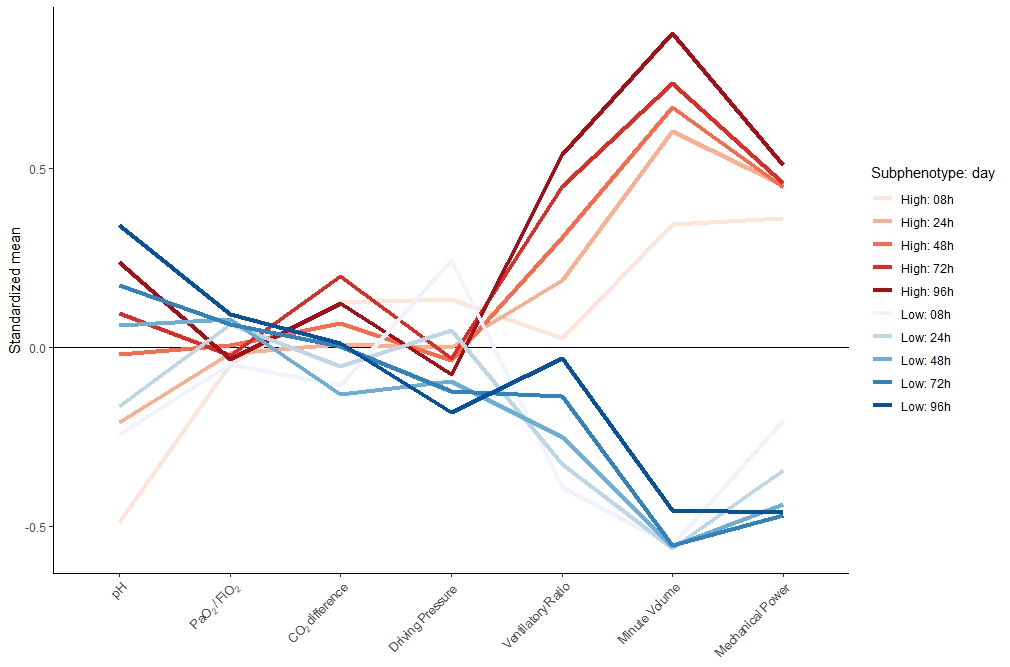


## Figure S5. Distribution of propensity scores


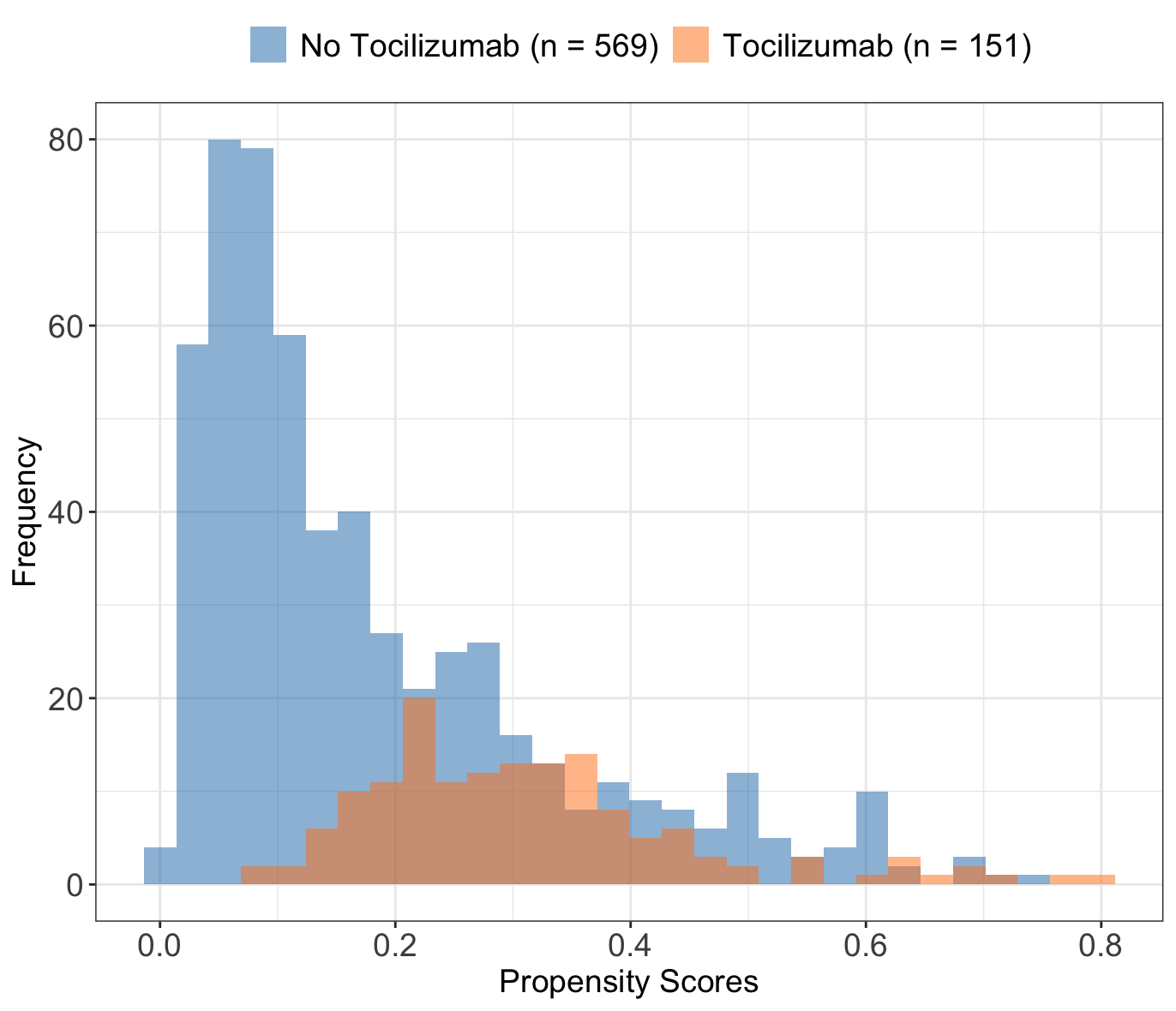

Propensity scores represent the probability of receiving tocilizumab. Propensity scores were calculated using a logistic regression model including age, sex, body mass index, medical history, vital signs at ICU presentation, laboratory measurements at ICU presentation, site and time period.

## Figure S6. Standardized mean differences before and after IPTW


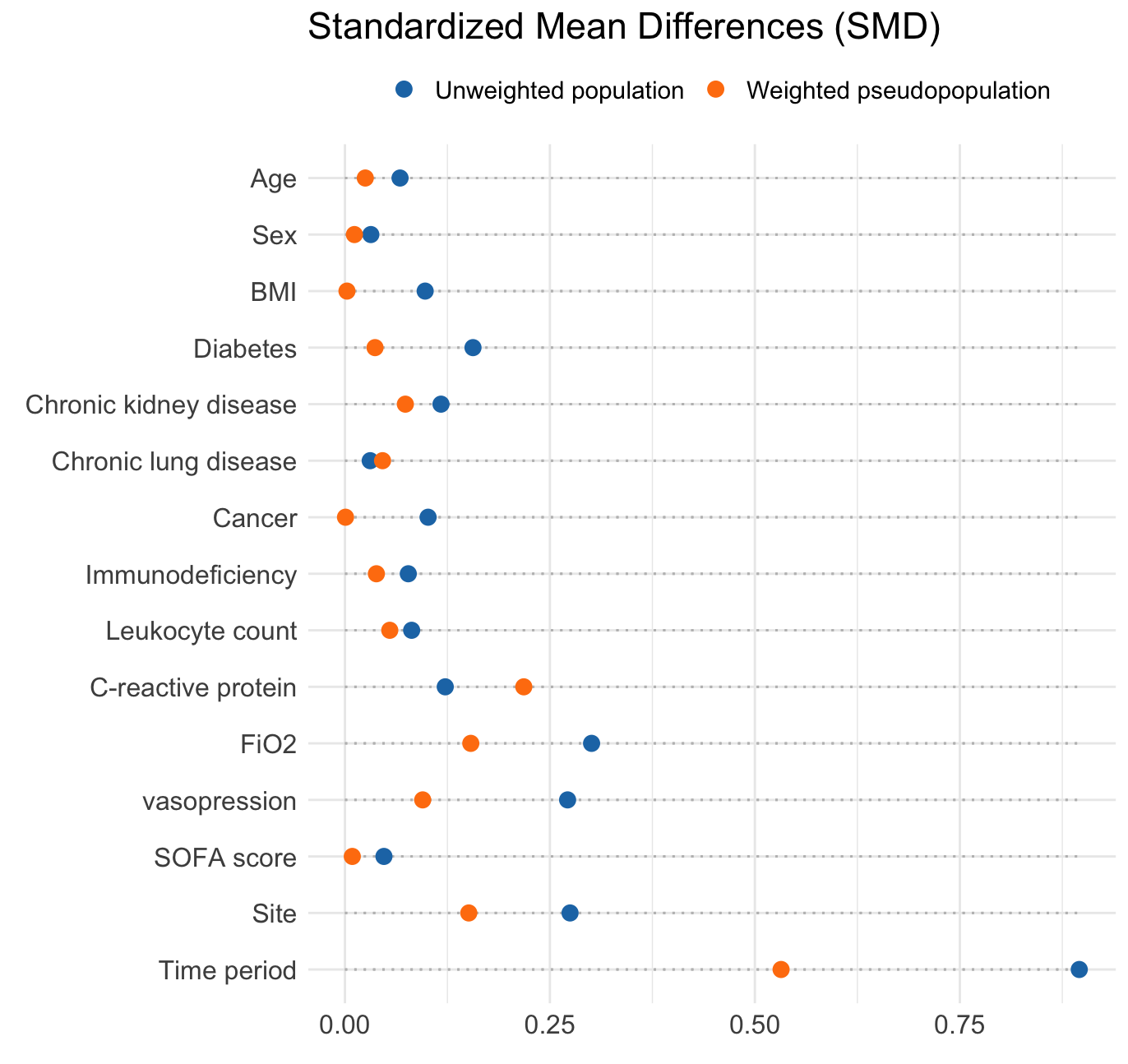


All variables presented were included in the logistic regression model that estimated the probability of receiving tocilizumab. Abbreviations: BMI = body mass index, SOFA = sequential organ failure assessment.

## Figure S7. Survival curves


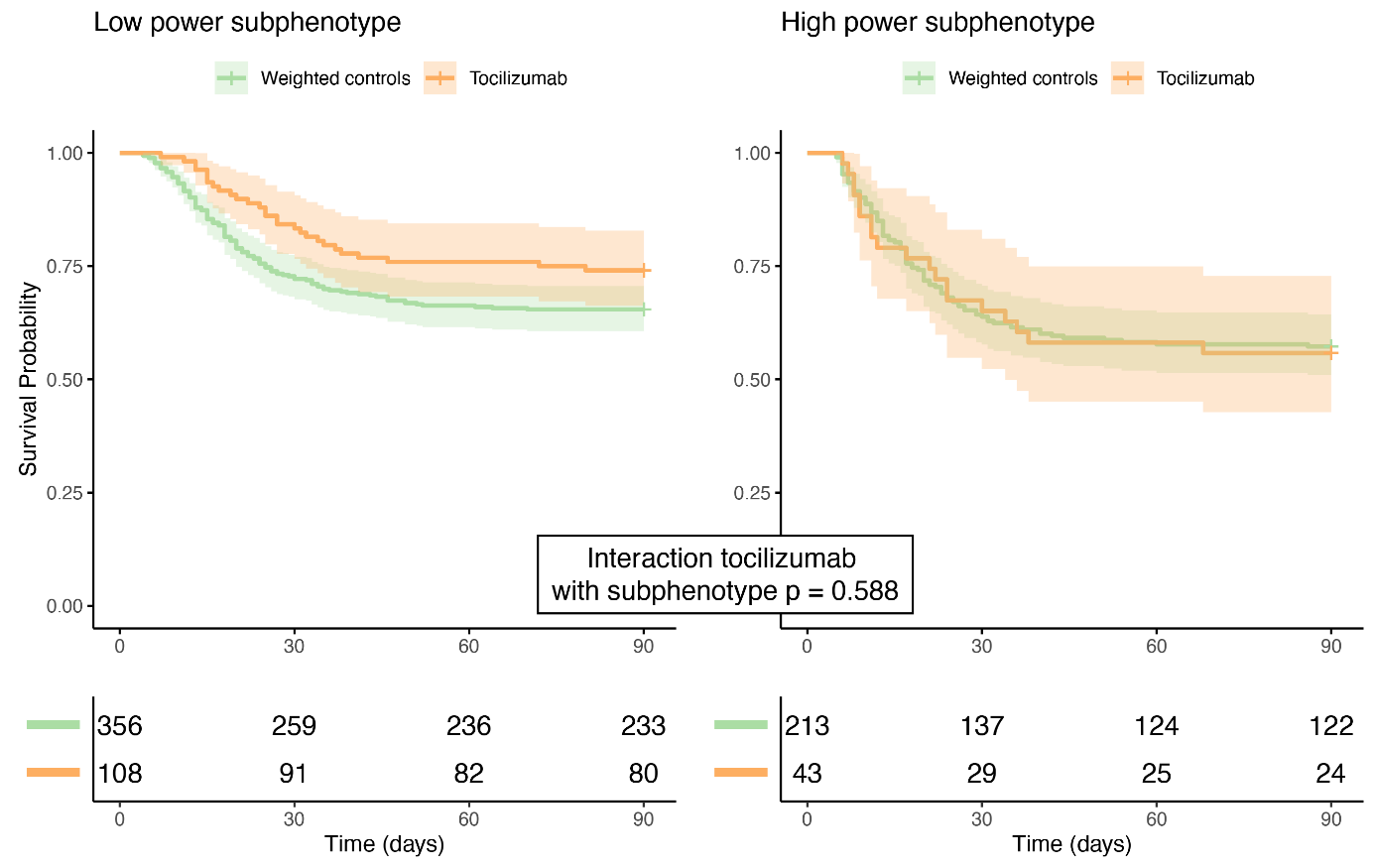


Abbreviations: adj. = Adjusted for age, sex, BMI, admission FiO_2_, admission Sequential Organ Failure Score (minus central nervous system score), admission C-reactive protein, site and time period. OR = Odds Ratio. *P* value of interaction between subphenotype and tocilizumab on outcome = 0.588.
